# A design thinking‐led approach to develop a responsive feeding intervention for Australian families vulnerable to food insecurity: Eat, Learn, Grow

**DOI:** 10.1111/hex.14051

**Published:** 2024-04-20

**Authors:** Kimberley A. Baxter, Jeremy Kerr, Smita Nambiar, Danielle Gallegos, Robyn A. Penny, Rachel Laws, Rebecca Byrne

**Affiliations:** ^1^ Centre for Childhood Nutrition Research, Faculty of Health Queensland University of Technology Brisbane Australia; ^2^ School of Exercise and Nutrition Sciences, Faculty of Health Queensland University of Technology Kelvin Grove Australia; ^3^ School of Design, Education and Social Justice, Faculty of Creative Industries Queensland University of Technology Kelvin Grove Australia; ^4^ Child Health Liaison, Children's Health Queensland Hospital and Health Service Brisbane Queensland Australia; ^5^ Institute for Physical Activity and Nutrition (IPAN), School of Exercise and Nutrition, Faculty of Health Sciences Deakin University Burwood Australia

**Keywords:** co‐design, design thinking, digital intervention, food insecurity, microlearning, parent, responsive feeding

## Abstract

**Background:**

Design thinking is an iterative process that innovates solutions through a person‐centric approach and is increasingly used across health contexts. The person‐centric approach lends itself to working with groups with complex needs. One such group is families experiencing economic hardship, who are vulnerable to food insecurity and face challenges with child feeding.

**Objective:**

This study describes the application of a design thinking framework, utilizing mixed methods, including co‐design, to develop a responsive child‐feeding intervention for Australian families—‘Eat, Learn, Grow’.

**Methods:**

Guided by the five stages of design thinking, which comprises empathizing, defining, ideating, prototyping, and testing. We engaged with parents/caregivers of a child aged 6 months to 3 years through co‐design workshops (*n* = 13), direct observation of mealtimes (*n* = 10), a cross‐sectional survey (*n* = 213) and semistructured interviews (*n* = 29). Findings across these methods were synthesized using affinity mapping to clarify the intervention parameters. Parent user testing (*n* = 12) was conducted online with intervention prototypes to determine acceptability and accessibility. A co‐design workshop with child health experts (*n* = 9) was then undertaken to review and co‐design content for the final intervention.

**Results:**

Through the design thinking process, an innovative digital child‐feeding intervention was created. This intervention utilized a mobile‐first design and consisted of a series of short and interactive modules that used a learning technology tool. The design is based on the concept of microlearning and responds to participants' preferences for visual, brief and plain language information accessed via a mobile phone. User testing sessions with parents and the expert co‐design workshop indicated that the intervention was highly acceptable.

**Conclusions:**

Design thinking encourages researchers to approach problems creatively and to design health interventions that align with participant needs. Applying mixed methods—including co‐design— within this framework allows for a better understanding of user contexts, preferences and priorities, ensuring solutions are more acceptable and likely to be engaged.

## 1 INTRODUCTION

The complexity of health behaviour calls for innovative approaches that respond to people's needs and contexts to improve programme engagement and outcomes. Despite the potential benefits of health interventions, they often fall short of their intended goals when implemented in real‐world settings.^1^ Traditional approaches to health programme development may be improved by adopting innovative, nonlinear, adaptive and cost‐effective tools.^2^, ^3^ Drawing from other sectors and disciplines offers potential within health to restructure our approach, enhancing interdisciplinary work across complex settings and allowing for diverse perspectives.^4^

Optimal child feeding requires a stable, nurturing environment that is responsive to children's health and nutritional needs, is emotionally supportive, and developmentally stimulating.^5^ The early years of life are a critical period for the formation of food preferences and eating behaviours that carry into adulthood.^6^, ^7^ Responsive feeding is a reciprocal process between caregiver and child in which the caregiver recognizes and responds appropriately to a child's hunger and satiety cues.^8^ Responsive feeding supports a child's intrinsic capacity to self‐regulate food intake.^9^ Through this mechanism, responsive feeding is considered protective against obesity risk, decreasing the prevalence of fussy eating and increasing fruit and vegetable intake.^9^, ^10^, ^11^ Nonresponsive feeding is characterized by a lack of reciprocity between caregiver and child.^8^ Adults may ignore, misinterpret, or not notice child cues. Parents play a crucial role in shaping their children's eating experiences^12^; interventions targeting parental feeding practices can support parents in this role.^13^

Parents experiencing economic hardship and food insecurity (FI) face additional child‐feeding challenges. FI is associated with poor health and is of particular concern in children. Household FI, even at marginal levels, has demonstrated impacts on children's behavioural, academic and emotional outcomes.^14^, ^15^ Food security exists when people have regular and reliable physical, social and economic access to sufficient safe, nutritious and culturally relevant food that meets their dietary needs and preferences.^16^ In high‐income countries, approximately 12% of individuals experience food insecurity,^17^ with higher rates in disadvantaged populations.^18^ In Australia, FI prevalence ranges between 4% and 36% depending on the measurement tool used and is experienced more in households with socioeconomic disadvantage.^19^, ^20^, ^21^ Low‐income families are more likely to experience household dysfunction and disorganization, impacting mealtime quality, routines, and child appetite self‐regulation.^22^ Food insecure households may experience barriers that impede responsive feeding and are linked to suboptimal parent–child interactions, including more controlling feeding styles.^23^, ^24^, ^25^, ^26^ This demonstrates the need for child‐feeding support among households vulnerable to FI that is contextually sensitive and perceptive to parents' experiences and needs.

Design thinking is a defined framework that integrates creative, interdisciplinary and person‐centred approaches to a topic or problem and is increasingly applied in social and health contexts.^27^, ^28^ A key feature of design thinking is the prioritization of deep empathy for end‐user desires, needs, and challenges to understand complex socioecological problems and to develop effective solutions that are contextually sensitive and viable.^28^, ^29^ Lockwood states that design thinking is ‘a human‐centred innovation process that emphasizes observation, collaboration, fast learning, visualization of ideas, rapid concept prototyping, and concurrent business analysis’.^30^ While design thinking can be undertaken from a human and user‐centred perspective, with a team designing *for* people, there is also an opportunity to apply it through a co‐design process. Co‐design is an approach that involves designing *with*—not for—the people affected by what is being designed.^31^ It entails creative methods to explore lived experience to inform designs, and the participants, acting as representatives of the user stakeholder groups, also develop ideas and concepts alongside the design team. Unlike traditional problem‐solving methods, design thinking prioritizes a deep understanding of the users' perspectives, enabling more empathetic and practical solutions; this may offer particular value in working with underserved populations whose needs may be overlooked by other approaches.^32^, ^33^

In this research, design thinking was adopted due to its human‐centred approach and potential to elevate the experiences of people experiencing disadvantage. Despite the increased use of design thinking in healthcare settings, there are limited applied descriptions of its adoption and critical components in health intervention design in the context of economic hardship or disadvantage.^32^ This paper aims to discuss and illustrate the application of a design thinking‐led framework using mixed methods in the development of a health intervention with an underserved population group. The outcome of this process was the ‘Eat, Learn, Grow’ intervention, which promotes responsive feeding practices among Australian families experiencing economic hardship and with a child aged 6–24 months.

## 2 METHODS

### 2.1 Research setting, recruitment and participants

The overarching research programme, Responsive Feeding in Tough Times, aimed to develop and evaluate an intervention supporting responsive feeding practices in families experiencing hardship and FI. The research team was based in Brisbane, Australia, and included expertise in dietetics, design, and child health nursing. Participants Australia‐wide contributed to the design thinking process. Given data collection occurred during the COVID‐19 pandemic (June 2021 to Dec 2022), remote methods (telephone, online surveys and videoconferencing) were adopted after challenges with initial face‐to‐face methods and in response to participant preferences.

The Children's Health Queensland Hospital and Health Service (CHQHHS) partnered in this research to engage with child health nursing services. Child health nurses play a pivotal role in Australia, providing universal free services for families of young children, including advice on feeding, health, and development. A research project partner within CHQHHS was appointed (R. A. P.) to facilitate communication between the university and health services partners.

To reflect the lived experience of families vulnerable to FI, recruitment methods were designed to identify caregivers who were economically struggling and caring for a child aged 6 months up to 3 years. A caregiver is any adult who provides regular home caregiving for a child; in this paper, ‘parent’ will be used throughout. Recruitment strategies for data collection included child health nursing and community organization stakeholders sharing recruitment callouts through parent networks. Social media posting, sponsored social media advertising, and snowball sampling were utilized. The screening question: ‘Do you sometimes struggle to pay the bills?’ was used to identify participants experiencing economic hardship. This screening question was established as an acceptable screening item with parent input. Informed consent was provided by those meeting the screening criteria.

### 2.2 The design thinking‐led approach

**Figure 1:**
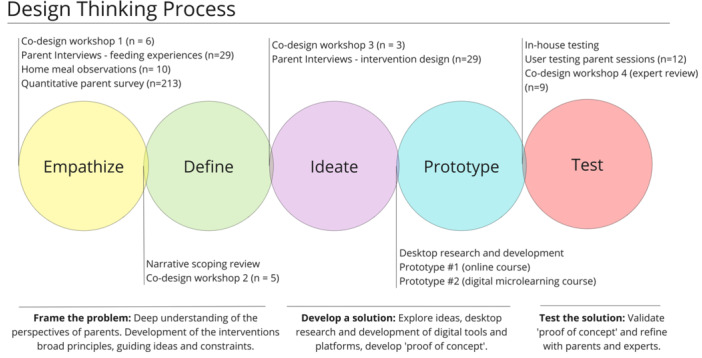
Overview and description of design thinking stages as applied to the intervention development.

The design thinking framework applied consists of five stages, each building on the output of proceeding stages in a nonlinear way, as proposed by the Hasso‐Plattner Institute of Design at Stanford.^34^ This allows innovative and responsive solutions to develop from five stages: ‘empathize’ (understand target population), ‘define’ (identify goals and scope), ‘ideate’ (brainstorm potential solutions), ‘prototype’ (develop mock‐ups of solutions) and ‘testing’ (gather feedback and refine).^35^ This project engaged parent stakeholders in the process, integrating co‐design elements. Mixed methods enabled a comprehensive understanding of stakeholder perspectives beyond those provided by quantitative or qualitative methods alone. An overview of the design thinking process and the actions undertaken are mapped to each stage in Figure 1. The following section summarises methods from each stage.

#### 2.2.1 Stage 1: Empathize

The objective of this stage was to understand parents' experiences; mixed methods were used to explore feeding practices and mealtime environments in the family setting. Co‐design workshop 1 was a 90‐minute face‐to‐face workshop (June 2021) that explored FI and informed recruitment strategies that were sensitive and nonstigmatizing. Activities prompted parents' perspectives on how to approach the topic of FI with families, what language to use and how, and where to recruit parents (see Appendix S1).

Parent interviews—feeding experiences were semistructured interviews that explored parents' experiences with feeding their families in the context of economic hardship and FI. These interviews were conducted to gather perspectives and experiences (August 2021 to January 2022) and included topics on family structure, mealtime setting and practices, food work responsibility and FI. Data were analyzed thematically using the framework analysis method.^36^ Detailed methodology and outcomes of these interviews are described elsewhere.^37^ Interview questions also included questions relating to the intervention design (i.e., Stage 3: Ideate).

A series of home mealtime observations were collected (August 2021 to February 2022). Parents were given a GoPro camera and asked to film three typical mealtimes at home with their child. The videos were transcribed verbatim and qualitatively analyzed against a coding framework that noted parent–child interactions and approaches during meals, mealtime environment and setting.

A cross‐sectional quantitative parent survey was completed (October 2021 to July 2022). Survey questions were developed to characterize feeding practices in families experiencing economic hardship and explore associations with FI and household dysfunction. Demographic data (income, education, ethnicity, family structure, housing mobility), child factors (age, gender) and household food responsibility were collected—validated tools measured FI,^38^ feeding practices,^39^ household organization and dysfunction^40^ and parental stress.^41^ Survey results and analysis were conducted using IBM SPSS Statistics.^42^

#### 2.2.2 Stage 2: Define

The second stage of the design thinking process, ‘define’, seeks to define the problem and the needs of those affected. This involved synthesizing the information collected from parents during the previous activities to articulate their needs and expectations for the intervention. Parents' ideas for a child feeding intervention from the ‘ideation’ phase also helped clarify the intervention's parameters.

A narrative scoping review systematically mapped existing literature regarding parental feeding practices in families experiencing disadvantage and FI in high‐income countries.^43^ Tools used to measure parental feeding practices and FI were described. Key features of interventions designed to modify parental feeding practices or styles among families experiencing disadvantage were catalogued. Methods and results of the scoping review have been previously reported.^43^

Co‐design workshop 2 focused on intervention planning (June 2021). The workshop explored family support networks, mapped sources of support and information on child health and feeding, and invited parents to share their experiences with these sources of support (see Appendix S1).

Analysis in the define stage occurred through iterative affinity mapping in Miro,^44^ where formative results, researcher notes and products from the co‐design workshops were visually collected and then grouped. Affinity mapping or diagramming is a common approach by user experience and design practitioners to sort findings.^45^ Affinity mapping is a process used to externalize and meaningfully cluster observations, data, and insights to help make sense of and organize unstructured data.^46^ This method was selected because of the mixed methods data and the need to synthesize ideas from multiple sources.

#### 2.2.3 Stage 3: Ideate

The ‘ideate’ stage involved generating ideas for the proposed intervention design, including content, delivery mode, structure and intensity. Co‐design workshop three was delivered face‐to‐face (July 2021), focused on intervention development, and explored intervention language and messaging from a successful feeding intervention, the NOURISH Trial.^47^ The NOURISH Trial recruited first‐time parents in Australia in 2008 and 2009. It provided anticipatory early feeding support to promote healthy weight and growth, embedded in a responsive feeding approach.^48^ Parents also participated in an unstructured brainstorming session to elicit ideas for the intervention design and features (see Appendix S1).

Parent interviews—intervention design included questions to validate and expand on the data from the co‐design workshop. The interviews explored where parents had previously accessed child health and feeding support. Parents' preferences and ideas on intervention mode, content and format were sought. The full interview methodology has been previously reported.^37^

#### 2.2.4 Stage 4: Prototype

In the fourth stage, ‘prototype’, we sought to respond to the intervention characteristics articulated in stage 2, considering parents' shared lived experience, and develop tangible models or ‘proof of concepts’ of possible solutions.

**Figure 2:**
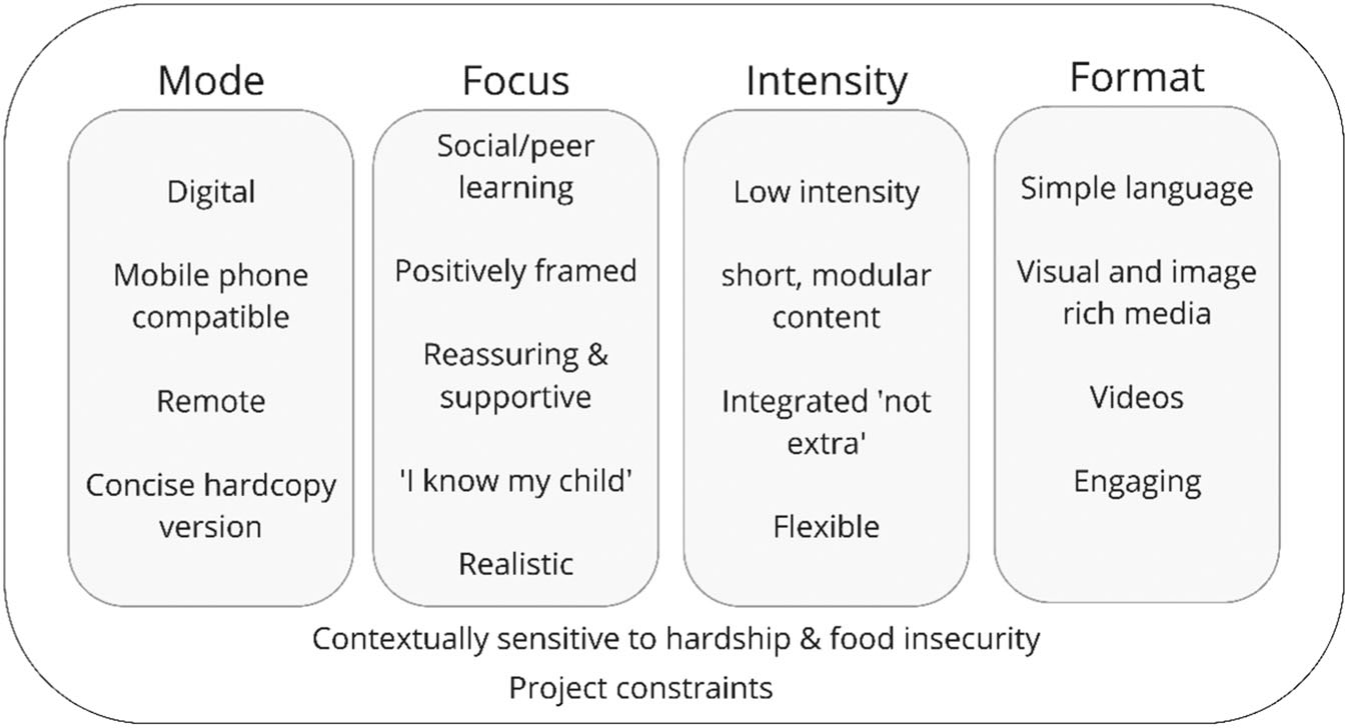
Broad principles of the intervention developed through affinity mapping.

Based on the outcome of the affinity mapping process (see Figure 2), a digital and remote mode of intervention delivery was explored with a printable or hardcopy version of key messages. This involved an initial desktop research and development phase, where potential digital platforms and web‐based applications were researched and trialled. Given project constraints of budget, timeline and research team skillset, no‐code applications or platforms that didn't require software engineering and development were selected. Two intervention formats were pursued: (1) an online parenting course and (2) a digital microlearning course.

*Prototype* 1: An online parenting course using Articulate 360, a web‐based application and content creator tool to design e‐learning courses for adult learners.^49^

*Prototype* 2: A digital microlearning course was pursued after further inquiry identified digital microlearning as better aligned with parent needs. A learning technology platform, 7taps was shortlisted as it was developed with a mobile‐first design.^50^ This ensured compatibility with the reduced dimension of a mobile phone screen and considered mobile users' needs. The platform enabled researchers to create mixed content, including videos, images, text and interactions, that could be delivered in a timed structure and collect user analytics. The content is web‐hosted, meaning parents don't need to download material, minimizing their data costs. Parent co‐design workshops and interviews in stages 1–3 informed the overall development of the intervention and the content.

#### 2.2.5 Stage 5: Test

In the ‘Test’ stage, feedback and testing were sought at three levels: (1) the research team and colleagues (in‐house testing), (2) parents and caregivers from the target group (user‐testing parent sessions), and (3) from child health experts and health professionals (co‐design workshop 4).

In‐house testing reviewed the proposed modes against the intervention parameters and refined the final intervention content through piloting. Prototypes 1 (online parenting course) and 2 (microlearning course) were reviewed in‐house before external review and feedback from parent stakeholders and health professionals. The final intervention was also piloted with the research team and additional colleagues to test the functionality of the interactions, analytics and the SMS delivery system with customized messages.

User‐testing parent sessions were completed (November ‐ December 2022) with three digital modules that would form part of a more extensive intervention (See Appendix S2). These were created using the 7taps platform and were designed to present different styles of videos, content and imagery to elicit feedback on the formats and parents' preferences. Web links to the modules were sent via SMS to participants' mobile phones on the day of the session. Parents viewed the content unmoderated. A video call was conducted on the same day to capture parents' impressions and feedback using Zoom videoconferencing.^51^ All video calls were recorded. During the sessions, the researcher shared a preview screen of the digital modules and guided parents through a talk‐a‐loud walkthrough. This was followed by open‐ended questions regarding the useability, accessibility, and satisfaction of the test modules. Parents' perspectives on recruitment and retention strategies, the language of key messages, the structure of the intervention and an activity to rename the parent programme were sought. Further details on the methodology of the user testing sessions have been reported previously.^52^

Co‐design Workshop 4 was undertaken after the user testing sessions confirmed the acceptability of the intervention; the course content was planned and expanded to include 12 microlearning modules (see Appendix S3). This content was reviewed by health professionals with expertise in child health, responsive feeding, and research (January 2023). This workshop included child health nurses, dietitians, and a psychologist. Health professionals engaged with the digital content as intended (i.e., on a mobile phone) to review language, videos, imagery and intervention structure. Participants were provided with an A3 book, which included a QR code for each module, screenshots of each ‘page’ of the content, and scripts for all audio and video material. Participants were asked to view the digital content on their mobile phones via the QR code and make notes and suggestions in the booklet and scripts. Key messaging and content were reviewed for alignment with a responsive feeding approach. There was a debriefing session at the end of the workshop to discuss and review feedback and share perspectives.

## 3 Results

### 3.1 Stage 1: Empathize

Co‐design workshop 1 (*n* = 6) gathered participants' perspectives on FI and informed recruitment strategies. Participants indicated that they wanted FI and hardship to be normalized and that it was a common experience within their family and social networks. Parents recommended material shouldn't have ‘tough times’ in the project name, feeling this was negative, and preferred reframing of FI to budgeting, managing and strategizing with limited resources. For recruitment strategies, parents indicated screening should be broadened to focus on economic hardship. The recruitment screening question, ‘Do you worry that food will run out before you are able to buy more?’ was changed as an outcome of the workshop. The screening question: ‘Do you sometimes struggle to pay the bills?’ was adopted, which was broader and considered a relatable Australian phrase. Incentives for research participation were viewed positively and as important for busy, low‐income parents. Participants suggested multiple strategies to approach parents, including social media advertising, parenting and mother groups, libraries, schools and other locations where parents frequent.

Parent interviews—feeding experiences were conducted with 29 parents across Australia. The demographic characteristics of participants are shown in Table 1. Thematic analysis generated five key themes: family tensions heightened through hardship, making trade‐offs and sacrifices, the unseen mental load, the inescapable impact of COVID‐19 and resiliency and being creative. Despite the challenges, parents demonstrated capabilities by enacting resilient systems of creative resource management to optimize food availability and quality in the home. Parents experienced a high mental load through the cognitive and emotional work of planning, adapting, anticipating, and caring for the family's needs through meals and child feeding.^37^ This informed the intervention by focusing on strengths and acknowledging parents as experts in their family with their own capabilities. Parents commonly described food‐related skills as essential to maximize family food resources. This was reflected in the intervention design by content focused on cooking, shopping, and meal planning, including the tips and suggestions from the parent interviews. The mental load experienced by parents was considered in the intervention choice and design.

**Table 1:** Demographic characteristics of participants involved in interviews, mealtime observations, quantitative survey and user testing sessions.

		Interviews (*N* = 29)	Mealtime observation (*N* = 10)	Survey^a^ (*N* = 213)	User testing (*N* = 12)
Mean age (range), years		32 (21−43)	30 (21–39)	31 (20–45)	30 (26−36)
Relationship status, *n* (%)	Partnered	24 (83)	6 (60)	187 (88)	10 (83)
	Single	5 (17)	4 (40)	25 (12)	2 (17)
Relationship to child, *n* (%)	Mother	28 (96)	10 (100)	207 (97)	10 (83)
	Father	1 (4)		2 (1)	2 (17)
	Other (foster parent/relative)			4 (2)	
Highest education, *n* (%)	Bachelor's degree	8 (27.5)	2 (20)	87 (41)	5 (42)
	Certificate/diploma	13 (45)	4 (40)	77 (36)	3 (25)
	High school	8 (27.5)	4 (40)	47 (23)	4 (33)
Income (AUD), *n* (%)	0–25,999 (0−499/week)	2 (7)	2 (20)	19 (9)	1 (8)
	26,000–51,999 (500−999/week)	9 (32)	4 (40)	56 (26)	1 (8)
	52,000−103,999 (1000−1999/week)	12 (41)	3 (30)	91 (43)	6 (50)
	104,000−155,999 (2000−2999/week)	5 (17)	1 (10)	35 (16)	3 (25)
	156,000 or more (3000+/week)	1 (3)		3 (1)	–
	Prefer not to say			9 (4)	1 (8)
Ethnicity, *n* (%)	Australian	22 (76)	7 (70)	150 (70)	9 (75)
	Aboriginal and/or Torres Strait Islander	2 (7)	2 (20)	2 (1)	1 (8)
	Other	5 (17)	1 (10)	61 (29)	2 (17)
Number of children, *n* (%)	One	1 (3)	2 (20)	106 (50)	4 (33)
	Two	6 (21)	2 (20)	65 (31)	5 (42)
	Three	9 (31)	5 (50)	26 (12)	1 (8)
	Four or more	13 (45)	1 (10)	13 (6)	2 (17)

^a^ Survey respondents: Relationship status: no response (*n* = 1; 0.5%); highest education: prefer not to say (*n* = 2; 1%); number of children: missing information (*n* = 3; 1%).

The home meal observations (*n* = 10) provided a window into family mealtimes, household environment, atmosphere, and practices. The small GoPro camera device was found to be unobtrusive and easy for parents to use; the characteristics of participants are shown in Table 1. Preliminary data analysis showed that feeding styles, practices and mealtime settings varied across families. Single‐child families typically showed calmer and less chaotic mealtimes, with more positive verbal interactions between the parent and child. Parental presence during mealtimes (i.e., at the table or within arm's reach) was associated with fewer behavioural problems during mealtimes and a calmer atmosphere. Mealtime distractions (e.g., pets, a television, toys or nearby conversations) appeared to interfere with mealtimes, where children would attune to these distractions rather than the meal itself; this flowed into observing more parental prompts to return the child's attention to eating. These findings informed the intervention content by including modules on family mealtimes, simplifying family mealtimes, and the importance of parent role modelling during eating.

The quantitative parent survey included 213 respondents, most of whom experienced low or very low food security (76%). Environments characterized by high levels of household chaos and FI were seen to impact parents’ ability to implement responsive feeding practices such as ‘food as a reward’, ‘feeding on demand’ and ‘food to calm’. The survey findings helped identify the feeding practices used more often among households that experience disadvantage and to develop content in line with these results.

### 3.2 Stage 2: Define

Formative findings were synthesized from the mixed methods approach in the ‘Define’ stage. Figure 2 reflects the intervention's broad principles from the affinity mapping analysis. The scoping review identified a lack of evidence to inform child feeding interventions that support disadvantaged families, including those with FI. The review recommended using validated tools to measure FI in at‐risk groups. In the interventions described in the review, a key feature was the high use of visual media, particularly videos. The review highlighted that cultural adaptation was essential when working with specific groups, and a strengths‐based approach targeting food‐related skills could be effective and well accepted by families experiencing economic hardship.

Co‐design workshop 2 (*n* = 5) was attended by all mothers; the activities were designed to explore where parents accessed child‐feeding support and to reflect on these sources to identify opportunities for the intervention design. Parents described mixed sources of child feeding and health advice with a preference for seeking health professional advice for health‐related issues and social sources for feeding‐related topics. Social support was accessed online and in person from family, friends, and mothers' groups; parents highly valued advice based on parental experience. Online and social media were discussed, with information accessed from credible online sources (e.g., government‐endorsed health or parenting websites) and less reliable social media groups and forums. Parents also highlighted their experience of conflicting advice from family members, which contradicted current health and feeding guidelines from health professionals and credible sources.

### 3.3 Stage 3: Ideate

Co‐design workshop 3 (*n* = 3) focussed on intervention development, content and design. Responsive feeding messaging and language were explored with parents to inform the intervention content and framing of key messages. Responsive feeding was generally well accepted and understood by parents, although parents were more aligned with this approach in the early years (birth—2 years), reporting a ‘firmer approach’ may be needed with older children. Some messages were considered unattainable for some families, such as eating together at mealtimes and repeatedly offering new foods to build a child's familiarity with them. They reported barriers such as a lack of time or insufficient food for everyone to eat together and the cost of wasted food as issues for food‐insecure families. The brainstorming session revealed parents' preference for digital and remote delivery, such as apps, short videos and remote modes. Parents also valued quick reference information and suggested a booklet with information tabs designed to be displayed in a prominent place, such as on the refrigerator using magnets. The final intervention incorporated many of these insights, including the quick reference booklet, digital learning, and videos. Responsive feeding messaging was also adjusted during content development.

In the interviews–intervention design (*n* = 29), parents described accessing advice and information from various sources with a preference for social support from friends, family, online sources and social media. Parents' experience of support on social media was mixed. This was usually based on previous experience, with some parents having negative interactions with other users. Instagram accounts that a health professional hosted were noted as credible sources of advice. Cooking, recipes, budgeting and strategies to manage reduced financial resources were common talking points. The mobile phone was where online support and information were accessed, with most not having a working home computer with internet. Online courses, digital programmes, apps and cooking shows were presented as ideas to provide intervention content. Time scarcity and information overload drove a need for messaging to be clear and concise.

Ideas and concepts generated from the interviews and co‐design workshop were synthesized via affinity mapping to refine and clarify the intervention parameters, summarised in Figure 2. Feedback from parents on key intervention messages in Workshop 3 helped shape intervention content and language around responsive feeding practices.

### 3.4 Stage 4: Prototype

The desktop research and development phase explored potential options against project constraints, including timeline, budget and research team skill set. Scoping of potential web‐based platforms and technologies to develop learning materials found several options for no‐code content creation tools. These were assessed against the intervention parameters (Figure 2) and considered requirements for data security, confidentiality, simplicity of design, and ease of use for both participants and researchers.

*Prototype* 1: an online parenting course using the web‐based application of Articulate 360 was explored. The online course was sketched out with potential modules, with one module developed as a mock‐up of the intervention. This was an introductory module on what is responsive feeding.

*Prototype* 2: a digital microlearning course using the learning technology tool 7taps was developed to explore the concept of microlearning. Three modules were developed: ‘What is responsive feeding?’; ‘Feeding roles—parent provides, child decides’ and ‘Learning about food away from the table’. Parent co‐design workshops and interviews in stages 1–3 informed the development of the intervention and the content of three test modules. The research team drew on an educational strategy called microlearning based on cognitive load theory to break important information into smaller, simpler pieces.^53^ This makes it easier for learners to process and retain information and concepts. Digital microlearning delivers clear and concise information to learners via digital modes such as email, SMS or scanning a QR code.^54^

**Figure 3:**
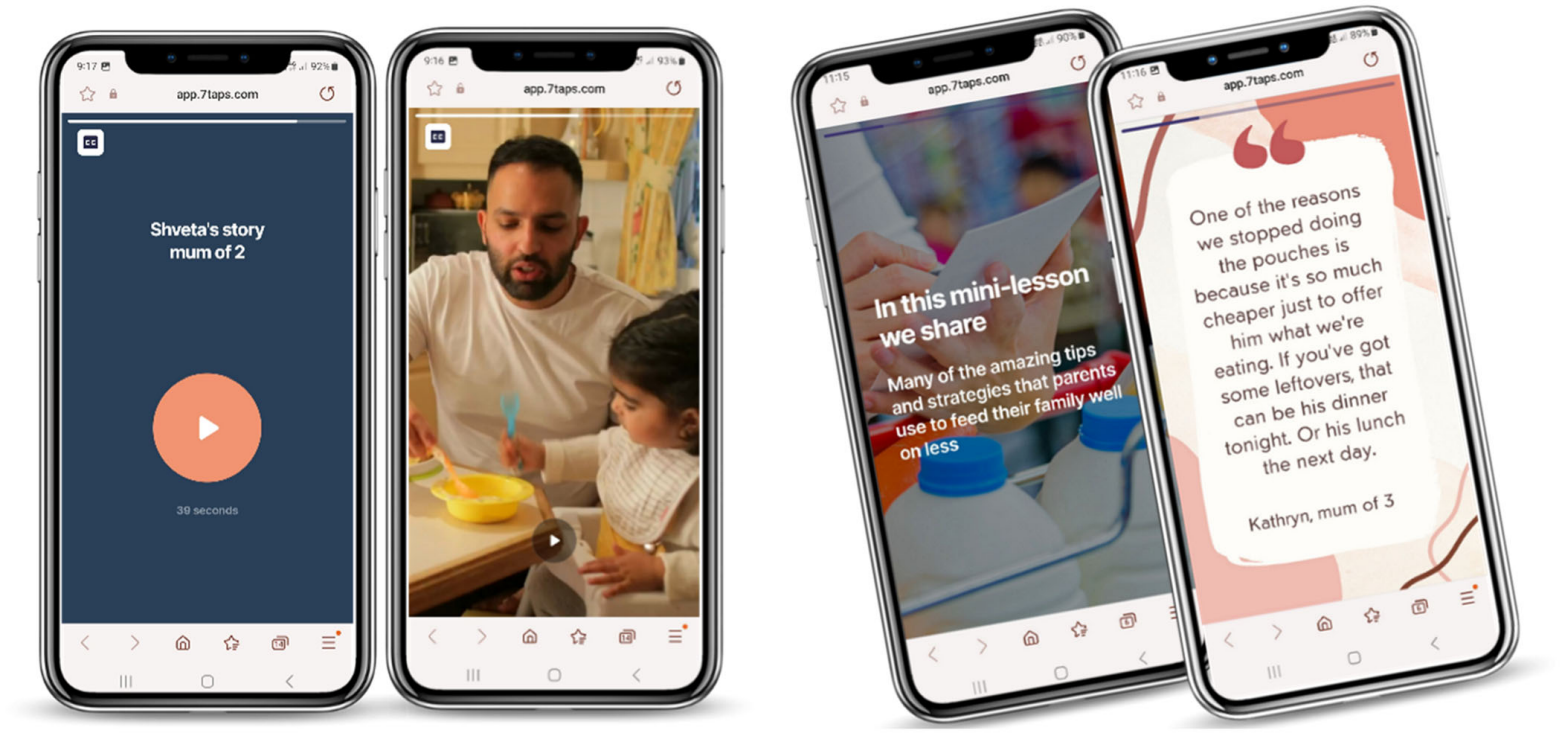
Screenshots represent content in the digital microlearning test modules, including parent quotes, audio stories and videos.

Informed by parents' input, the content used principles of social learning and storytelling with parent quotes and audio ‘stories’ from interview excerpts. These reflected the experiences of real families and supported key messaging, see Figure 3.

Engagement found a portion of parents who wanted hardcopy materials as part of a child‐feeding intervention. This material would be in the home kitchen space and act as a quick reference with gloss paper to be water resistant. This resulted in a physical mailout package of resources being designed to complement the primary content of the microlearning course. The mailout pack items were informed by the scoping review, interviews and co‐design workshops and, as part of the prototype development, were presented to participants as mock‐ups.

### 3.5 Stage 5: Test

Feedback was gathered on the intervention iteratively to refine and finalize the design and content. In‐house testing within the team and colleagues helped guide the process and reflect on findings from the engagement with parents over Stages 1–3. As a result, the development of Prototype #1 (online parenting course) intervention mode was halted. The platform was not adequately mobile phone compatible and relied on written text, with fewer options for integrating audio and video content.

The user‐testing parent sessions (*n* = 12) found that participants reported the content was accessible, with the self‐paced nature enabling them to move through and revisit the content as needed. Three parents voluntarily reported being neurodiverse, which impacted their ability to learn and process information (e.g., attention deficit hyperactivity disorder, dyslexia, aphantasia). Parents found the content intuitive to move through and enjoyed the mixed content of text, videos, images and interactions. The parent quotes and short audio stories were well‐liked, making the content relatable and nonthreatening. The short videos were highly acceptable. Including closed captions for all audio content was indicated to be helpful for understanding audio information. However, parents reported that the modules were too brief and should be expanded.

Key outcomes from user testing included adding more parent quotes, stories from the interview transcripts, and videos. The content was also expanded to include more details and examples. The proposed structure of the microlearning course was acceptable to parents. The content of the mailout pack was well‐liked, and no changes were made. Finally, the name of the parent intervention component of the Responsive Feeding in Tough Times project was changed to ‘Eat, Learn, Grow’.

Co‐design Workshop 4—expert review (*n* = 9) consisted of a multidisciplinary review of the intervention, including content, structure, design, language and visual imagery. Participants indicated the programme's acceptability and thought the novel style of parenting support would have high resonance with the target group. Key recommendations were to strengthen the behaviour change strategies, with clearer wording for planning behaviour changes, a consistent format of the modules and to extend the time the modules are delivered from 4 to 6 weeks to reduce the load on parents. Refinement of language and identification of inconsistencies across the modules were also noted. These changes were reviewed and, where possible, incorporated into the content.

The key formative findings that emerged from each stage of the design thinking process and contributed to the final design are summarized in Table 2. These findings represent a building understanding of the participant group's experiences and needs, which informed the intervention mode, design and content.

**Table 2:** Findings from mixed methods across the design thinking stages.

Empathize	Define	Ideate	Prototype	Test
Parents experience a high mental load. Families develop resilient systems to manage resources and optimize mealtimes. Parents prefer hardship and food insecurity to be framed as ‘budgeting’. A strength‐based approach will be well‐received. Parents feel information overloaded.	Video and image‐rich media is preferred. Research is lacking to inform feeding interventions. Families work to develop support networks. Parents value experiential stories and advice (from other parents). Mobile phones are a hub for child health and feeding information. ‘I know my child’.	Digital and remote support is preferred. Some parents value hardcopy resources in addition to digital. A practical focus is important. Mobile phone compatible. Self‐paced and modular. Visual and engaging: ‘can you do Insta stories?’ The ‘feel’ is important: empowering, reassuring and nonjudgemental.	Remote delivery offers value and benefits. Project constraints dictate ‘off the shelf’ technology and no code development. Mobile first design. A mix of media content is important (videos, images, interactions, text).	Well‐liked and acceptable. Self‐paced content supports learning. The programme name was changed to: ‘Eat, Learn, Grow’. Expert‐led. Behaviour change focus needed reinforcement. Use a consistent format for modules, including more interactive activities. Intervention structure to be extended (4 weeks ‐> 6 weeks). Language refined; errors/inconsistencies identified.

### 3.6 Description of the intervention: ‘Eat, Learn, Grow’

The final intervention is a parent‐focused digital microlearning course of modular content informed by the responsive feeding framework, as described by Perez‐Escamilla et al.^55^ A mail‐out package of resources accompanied the microlearning course. The intervention is delivered remotely and includes twelve digital modules of 3–5 min duration sent via SMS over 6 weeks. A summary of the microlearning ‘Eat, Learn, Grow’ programme content is shown in Table 3, including the learning objective of each module, key messaging and strategies used.

**Table 3:** Overview of ‘Eat, Learn, Grow’ digital microlearning course—objectives, content, strategies.

Module title	Module objective	Content	Strategies
Welcome	Welcome to the programme, set the context and familiarise with digital mode and programme structure.	Context of responsive feeding (RF) Introduce the team (video) and programme	***Knowledge provision:*** Clear, nonjudgemental, factual curriculum designed with microlearning principles. ***Shared learning:*** Digital note boards (padlets) ***Knowledge testing:*** Quizzes Polls Short form questions ***Reflection:*** Reflect on past and current feeding practices ***Reinforcement:*** Cross‐reference to components of the intervention to reinforce messaging Use of consistent terms and language Recap of key messaging ***Behaviour modelling*** ***Video demonstration of feeding practice*** ***Peer modelling:*** Short stories and quotes from parents to support content messaging—behaviour‐focused. ***Call to action*** ***Calls to implement key messaging into practice***
What is responsive feeding?	To recognize RF and the key principles of this approach.	Babies are born with the capacity to self‐regulate food intake Definition of responsive feeding Why use an RF approach Common hunger and fullness cues	
Responsive feeding: What does it look like?	To apply responsive feeding practices and strategies in mealtime settings and feeding situations with their child.	Metaphor of RF is a conversation (back and forth) Feeding structure to support RF Video of hunger and fullness cues and parent responses	
Feeding roles: Parent provides, child decides	Identify the role of the parent and the child based on Ellyn Satter's Division of Responsibility in Feeding and how to apply this in feeding situations.	Definition of feeding roles (parent and child) under the division of responsibility Eating is a learning process	
Learning to eat takes time	Understand that eating is a learning process and apply evidence‐based practices to support food acceptance with their child.	Food refusal is normal Strategies to support food acceptance, e.g., offering food multiple times without pressure Food wastage can be of concern	
Mealtimes are about more than food	To identify what a family meal is, the benefits of regular family meals, and apply strategies to do them more often.	Family meals don't have to be perfect Benefits of regular family mealtimes for parents and children extend beyond eating Tips for regular mealtimes	
Let's eat together: Role modelling	To appreciate the vital role of parental modelling in child feeding, encourage parents to model healthy eating and plan more opportunities to role model with their child.	What is role modelling? Why role modelling is important How parental role modelling of eating impacts child eating behaviour	
Family food talk	Establish positive feeding strategies and food language to promote food acceptance and reduce food conflict at mealtimes.	Food is central to many aspects of life (culture, celebration) Parents sometimes use food to control child behaviour (rewards, bribes) Neutral approach to food	
Learning about food away from the table	To appreciate that children experience food through their senses, which increases food familiarity; To scaffold more opportunities for their child to explore food.	Children learn about food both at the table (eating) and away from the table (playing) Ideas to support young children's growing experience with food Learning about food can be messy	
Variety is the spice of life	To apply the concept of food variety to child feeding and use strategies to increase food variety.	Why is variety important? What is food variety? Simple strategies to offer child more variety	
Being creative	To share the experiences and tips for feeding the family on a budget from other parents.	Being creative with food resources can help reduce costs and stress Meal planning Tips and strategies from parents	
What's next…	To acknowledge parents' contribution, revise key messages, deliver the T1 survey.	Reinforce key messaging Link to T1 survey	

**Figure 4:**
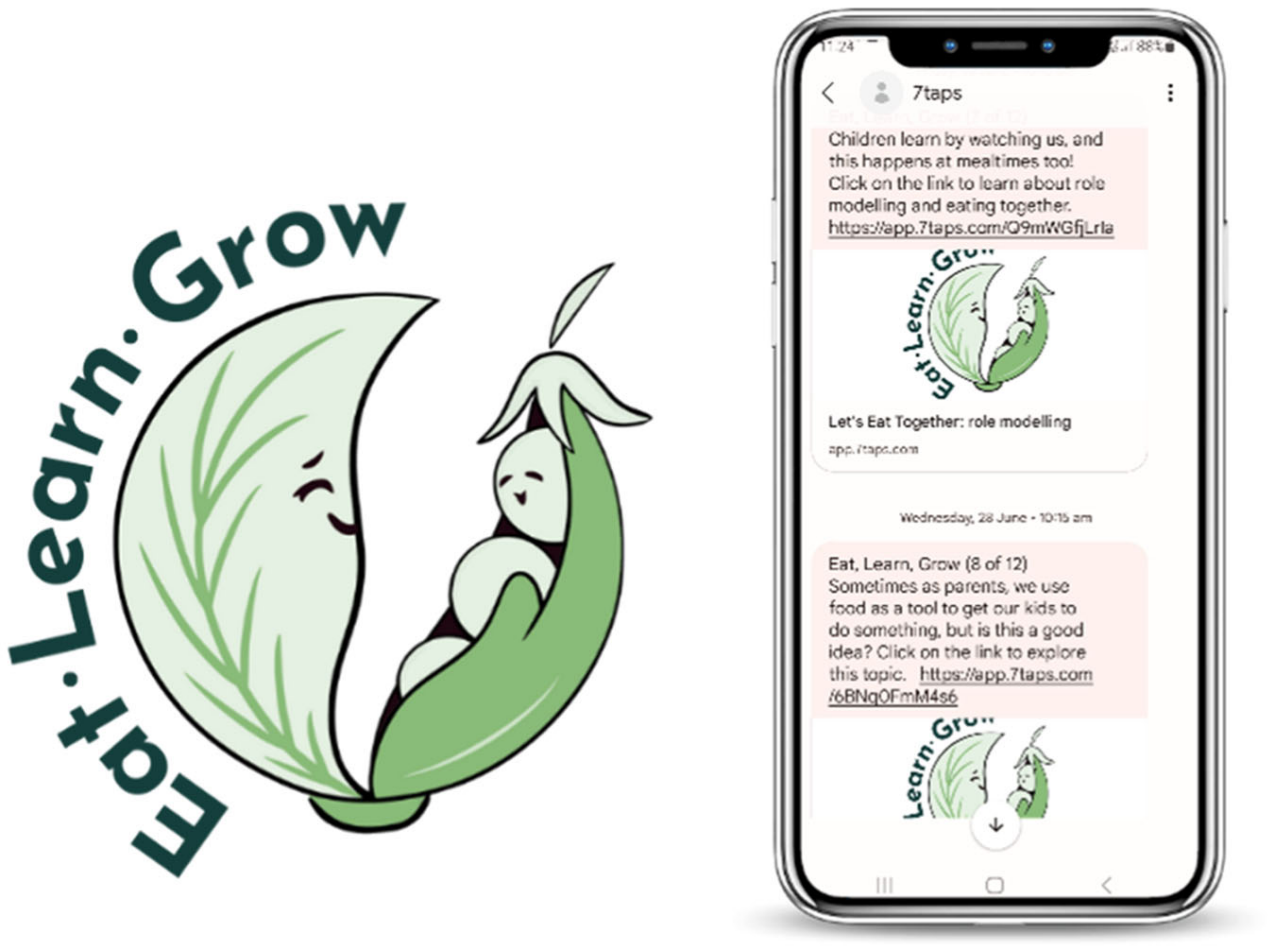
Rename and logo of parent‐facing intervention and SMS delivery system featuring logo.

As indicated in stage 1, participants wanted hardship and the experience of FI to be normalized and framed positively. In response, the intervention name was changed from ‘Responsive Feeding in Tough Times’ to ‘Eat, Learn, Grow’, with parents' input. A logo was designed to create consistency and build trust with the content web links, Figure 4. This was important given the general concern of spam and unsolicited text messages and links.

**Figure 5:**
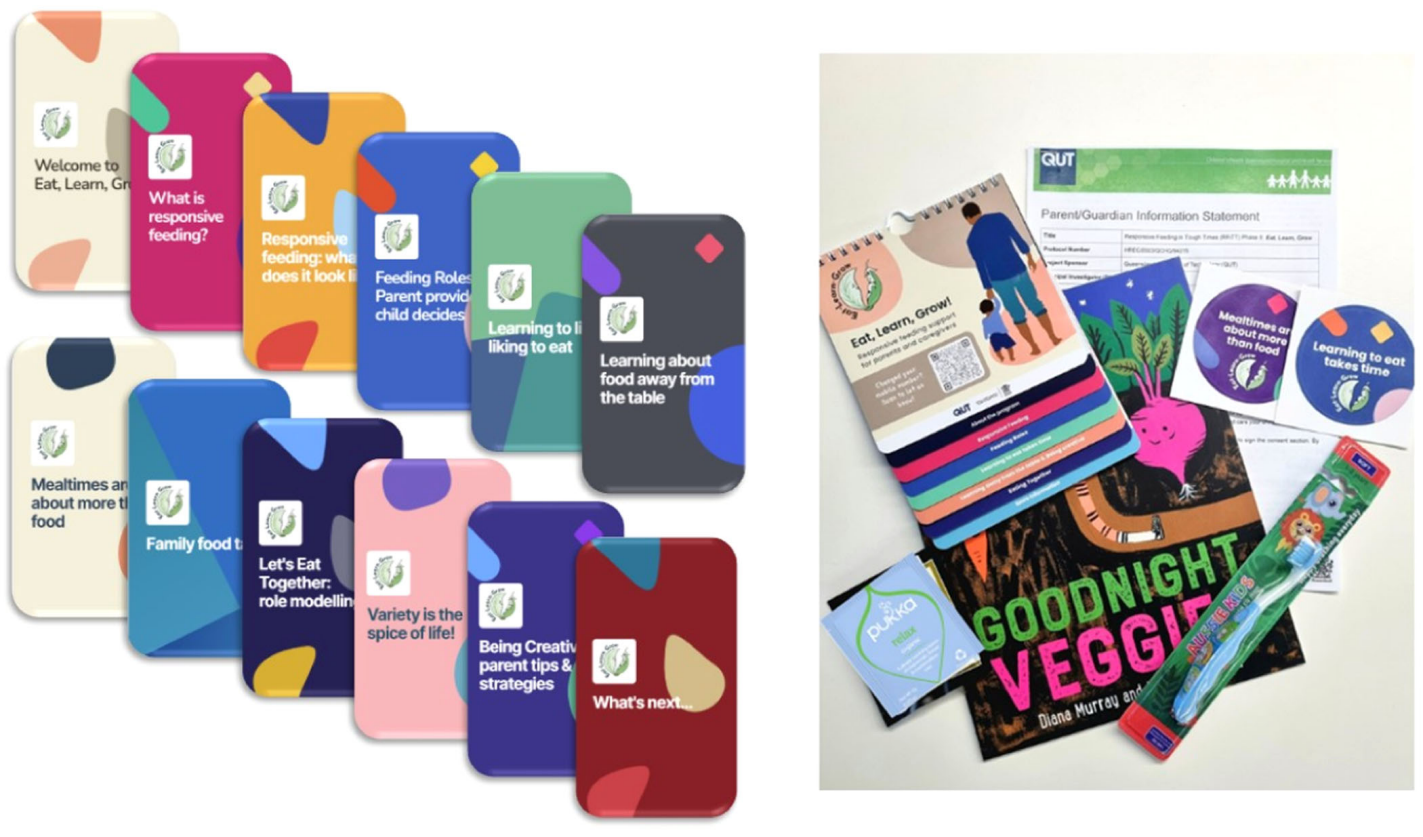
The ‘Eat, Learn, Grow’ programme includes 12 microlearning modules and mailout packages.

A mailout package included a hardcopy resource booklet with the study logo and key intervention messaging to reinforce the digital content. The booklet had magnets and was designed to be placed on the fridge in the family kitchen. The resource had QR codes linked to parenting topics from credible web‐based sources. Topics were informed by parent engagement and included food allergy, general parenting, oral health, choking first‐aid, introduction to solids and cooking on a budget. Other items in the package included a baby toothbrush, a children's storybook with an underlying food literacy message, stickers with intervention messaging and two teabags. The ‘Eat, Learn, Grow’ intervention materials, including the mailout package and indicative screenshots of the 12 microlearning modules, are illustrated in Figure 5.

## 4 DISCUSSION

This paper has described and illustrated a design thinking‐led approach using mixed methods to develop a parenting intervention among an underserved population in Australia. This person‐centred focus enabled co‐design at meaningful points with the target group: families with young children experiencing financial hardship and vulnerable to FI. The resulting intervention, ‘Eat, Learn, Grow’, has been successfully developed and is currently being evaluated in a randomized controlled trial (ACTRN12623000513617). The trial will assess the impact on parent feeding practices and the acceptability of the digital microlearning mode. The ‘Eat, Learn, Grow’ programme is an innovative, responsive approach to a complex problem, leveraging learning technology that has yet to be used in this context. It can provide insight into the potential use of digital microlearning across other health promotion contexts and in the development of interventions in the parenting space.

A strength of this work was the meaningful engagement and contribution from parent stakeholders through the iterative design thinking approach. Deep engagement with and understanding of the target population ensured that parent experiences were represented in the design of the intervention. This aligns with best practice intervention development, which calls for person‐based approaches and engagement beyond consultation and acceptability testing.^56^, ^57^, ^58^ Design thinking involves the holistic interpretation of data, not just the data output itself.^59^ Genuine engagement with parents identified the high mental load experienced in terms of the cognitive and emotional load of caring for the family and the persistent need to be resilient in the face of adversity.^37^ This pivotal point of empathy shaped the intervention design to use digital microlearning, drawing on cognitive load theory. This may have been missed using other frameworks that do not include this empathy stage. Also identified from the process was the high‐value parents placed on experiential knowledge. The intervention reflected this through quotes and short audio stories from the interview transcripts to support and contextualise the messaging provided. Using social context in interventions targeting feeding practices among families with a lower socioeconomic position was a key recommendation from Larsen et al.^60^ This research has demonstrated that creative and innovative approaches can meet the needs of parents with complex needs.

Design thinking offers value when engaging with underserved populations and disadvantaged communities.^32^ Efforts should be directed toward establishing deep trust and rapport through engagement, particularly those in underrepresented groups, to ensure the applicability and impact of research findings and end products.^61^ Design thinking may help address this by recognizing the inherent knowledge base of end‐user groups.^62^ A design thinking framework allows for the integration of co‐design at meaningful points. This is especially helpful when projects do not have the resources or opportunity to engage co‐design throughout a project. Previous research has successfully incorporated design thinking to develop health interventions across various areas.^63^, ^64^, ^65^, ^66^ Of note in this research is the mixed method approach, which incorporates literature, quantitative and qualitative approaches in developing the Eat Learn Grow programme and is underpinned by the responsive feeding framework.^55^ Woods et al. reported a similar approach in designing a mobile health app for heart failure, noting this as a key strength of their research.^65^ Frameworks have been put forward that apply design thinking principles to the development of health‐related digital technologies or programmes; some examples include the IDEAs framework,^67^ LAUNCH^68^, ^69^ and behavioural design thinking for mobile health interventions.^58^ These types of integrated frameworks may provide nondesigners, such as health professionals, with an easy‐to‐apply process that can be used in a range of settings. One of these integrated frameworks^58^, ^67^, ^68^, ^69^ may have been well aligned to the development of ‘Eat, Learn, Grow’. However, in conducting this project, the digital mode of intervention was not determined from the outset, but rather identified through the user‐centred approach. Research projects seeking to apply design thinking principles to digital health projects may find these models useful and report on their applicability across various settings to contribute to knowledge in this field.

Adopting the design thinking framework in this project produced unexpected benefits through its agile and responsive approach to the changing COVID‐19 situation at the time. Remote data collection methods align with design thinking and were adopted to alleviate recruitment difficulties and meet parent preferences. This pushed recruitment outside the original scope to be Australia‐wide, resulting in the developed programme having applications in the broader Australian context. Remote methods also benefited participants by removing barriers to research participation among disadvantaged groups,^61^ including time, travel, caregiving, and work responsibilities. Remote research methods can increase anonymity and safety, breaking down power discrepancies between researchers and community participants from underrepresented groups.^52^ Participants in this study readily accepted remote methods for the interviews (telephone; August 2021 to January 2022) and user testing sessions (video call; October–December 2022), reporting increased familiarity and confidence with remote methods, having used telehealth services, and working from home during the COVID‐19 pandemic. However, COVID‐19 introduced challenges for engagement with child health nurses and health professionals, which could not be overcome through agile and remote methods. The intention was to conduct early co‐design workshops with nurses/health professionals, but the significant and unprecedented strain on the health system from the response to COVID‐19 made this unworkable. The final co‐design workshop with child health experts ensured validation and review of the intervention's final content, structure, and evaluation.

## 5 LIMITATIONS

This study has limitations that require consideration. The COVID‐19 pandemic and strategies to reduce community transmission of COVID‐19 had a multifaceted influence on research in Australia and worldwide. COVID‐19 may have influenced parents’ responses and contributed to their preference for remote and digital modes. However, parents' use of and preference for digital support and programmes in child health and parenting has been reported in other studies.^70^, ^71^

This study carefully considered recruitment strategies to engage parents experiencing economic hardship. However, only a small proportion of parents from Aboriginal and Torres Strait Islander and culturally and linguistically diverse families or fathers were represented in this research. The data collection methods and resulting intervention design also excluded parents without a mobile phone or internet, which may include those experiencing homelessness, domestic and family violence or housing insecurity. Further research and engagement methods should explore how best to reach these participant groups.

## 6 CONCLUSION

This paper illustrates the feasibility and value of design thinking when applied to a complex health problem with an underrepresented population group. Utilizing mixed methods—including co‐design at critical points within this framework allows for a better understanding of user contexts, preferences and priorities, ensuring solutions are acceptable. This meaningful engagement with parents led to an innovative digital intervention that represented parents' experiences and addressed their needs. Design thinking encourages researchers to approach problems creatively and should be overlaid with theoretical frameworks that support behavioural health interventions.

## AUTHOR CONTRIBUTIONS

**Kimberley A. Baxter**: Conceptualization; investigation; writing—original draft; methodology; writing—review and editing; project administration; data curation; formal analysis. **Jeremy Kerr**: Conceptualization; writing—review and editing; methodology. **Smita Nambiar**: Conceptualization; funding acquisition; writing—review and editing; methodology; formal analysis. **Danielle Gallegos**: Conceptualization; writing—review and editing; funding acquisition; formal analysis; methodology. **Robyn A. Penny**: Conceptualization; writing—review and editing; formal analysis. **Rachel Laws**: Conceptualization; writing—review and editing. **Rebecca Byrne**: Conceptualization; funding acquisition; methodology; writing—review and editing; project administration; investigation; formal analysis.

## CONFLICT OF INTEREST STATEMENT

The authors declare no conflict of interest.

## ETHICS STATEMENT

The CHQHHS Human Research Ethics Committee (HREC) (LNR/21/QCHQ/72314) and the Queensland University of Technology HREC (2021000193) provided ethical approvals. The CHQHHS Research Governance Office granted site‐specific approval.

## Supporting information

Supporting information.

Supporting information.

Supporting information.

## Data Availability

The data that support the findings of this study are available from the corresponding author upon reasonable request.
