# Supplementary material for: A design thinking‐led approach to develop a responsive feeding intervention for Australian families vulnerable to food insecurity: Eat, Learn, Grow
Source: Health Expect. 2024 Apr 20;27(2):e14051. doi: 10.1111/hex.14051 (PMC11032130; doi:10.1111/hex.14051)
Supplement: Supplementary file 1 — Supporting information. [file HEX-27-e14051-s002.docx]

# Appendix 2: The Responsive Feeding in Tough Times Project: User Testing Session protocol and guide

**Remote user-testing sessions – 1:1 with parents**

**Resources:**

- Complete online consent process
- SMS the 3x module links on the arranged day
- Slides for sharing
- IT resources for Zoom session
- 7taps platform preview screen of modules
- Electronic gift vouchers

**Activity Overview:**

- [before session] Parent engages with the 3x digital modules
  - Links to the modules sent via SMS to their mobile phone device.
- Useability Feedback
- Talk-a-loud walkthrough of the 3 digital modules
- Parent Feedback
  - Invite parents to share their perspectives on key language and proposed program timing, structure, and delivery.
- Wordplay activity
  - brainstorm a new name for the program

**Welcome and introductions—**This session is designed to gather your valuable feedback on the usability and content of the 3x digital modules. We aim to use your insights to improve the modules and enhance the program delivery.

**Activity 1: Feedback on Usability**

Can you tell me how you found opening the links and moving through the content on your mobile phone?

- Did you have any trouble opening the link to the digital modules?
- Did you have any connection issues or a slow connection?
- How did you find moving through the content?
- Did any images, text, audio, or videos not work well when you viewed the lessons, were misshaped, or off-screen?

**Activity 2:** **Talk-a-loud walkthrough**

Invite the parent to share general impressions and thoughts on the content viewed prior.

[share screen on the 7taps preview screen and move through the content with the parent]

- Invite the parent to share their comments/impressions and feedback on the content ‘slide by slide’ for each of the three modules

[allow the participant to speak on the content – ask questions where clarification is needed]

- After going through the 3 modules, prompt the participant to comment on:
  - The different styles of videos included (i.e., macro view of child behavior vs expert speaking vs animated graphics)
  - Imagery
  - Least liked / most liked parts of the content
- How engaging did you think the modules were?
  - Rate on a scale of 1 (not at all) to 5 (very engaging)
  - Why did you choose that number?
- What did you think about the length of the modules?
  - Too short, too long, just right
- What did you think about the different formats used through the modules?
  - Text, audio, video, parent quotes, parent stories, quiz, digital note board link
  - Invite feedback into each format - what did you like/didn’t like;
  - What did you think about the balance of the different formats?
  - What would you have liked more/less of?

**Activity 3: Parent Feedback on Language & Program Structure**

[ask for their thoughts on the words used in the content and to suggest any alternatives they’d prefer; after they’ve exhausted their ideas, offer the options listed below]

- *‘*parent’
  - Options = parent, adult, caregiver, carer
- ‘baby’
  - Options = child, infant, baby, toddler
  - Should we interchange these words or be consistent throughout?
- ‘cues’
  - *Much of the idea behind our resource is about the communication between the adult and child during feeding – those verbal and non-verbal communication signs. What do you think these should be called?*
  - Options = cues, signs, signals
- ‘resource’
  - What should we call this program?
  - Options = resource, program, intervention, micro-learning course
- ‘lesson’
  - What should we call the digital modules?
  - Options = lesson, module, e-lesson, micro-lesson, mini-lesson, digital lesson, Digi-lesson, message
- ‘Low income’
  - *This program is designed with families in mind who are struggling to pay the bills and may have concerns about being able to buy enough food to feed their families. What is the best word to describe families in this situation?*
  - Options = Low income, tough times, doing it tough, budget/budgeting,

Describe the proposed mode of delivery and structure of the program (SMS of 12 modules over four weeks)

- What do you think about this schedule?
- Would you change it? If so, how?
- Is there a particular time of day we should send out the modules?
- Give an overview of the proposed mailout package and invite parents' feedback.

**Activity 4: Brainstorm – program name**

Our parent program is currently called ‘Responsive Feeding in Tough Times.’

- What do you think about this name for the program?

Other parents who have participated in this project have told us that this name is too long and could be framed more positively. What do you think?

- Do you have any suggestions for a new name
- Share the screen of the slide with program themes– invite the parent to ‘play with words’, to try rhyming and to suggest new words [add to slide between each session to build on the program name]

**Final thoughts**

*Do you have any further thoughts or suggestions about the digital modules or anything else we’ve discussed today?*

**Wrap up, summarize key talking points from the session and invite comments.**

**Thank you!**
