# Supplementary material for: A design thinking‐led approach to develop a responsive feeding intervention for Australian families vulnerable to food insecurity: Eat, Learn, Grow
Source: Health Expect. 2024 Apr 20;27(2):e14051. doi: 10.1111/hex.14051 (PMC11032130; doi:10.1111/hex.14051)
Supplement: Supplementary file 2 — Supporting information. [file HEX-27-e14051-s001.docx]

# Appendix 1: The Responsive Feeding in Tough Times Project: Codesign workshop protocol and guide – Parent workshop series (3 of 3)

**Workshop 1: Food Insecurity & Recruitment Strategies**

**Resources:**

- Consent forms
- pens and markers
- facilitator checklist – admin
- Large paper sheets
- workshop guide
- Individually wrapped snack items
- Thank you vouchers

**Activity Overview:**

- Food-related everyday decision map (#1 – group)
- Food-related decision map (#2 - family)
- When times are good/hard
- Barriers and enablers – add coded green, red, or yellow
- What makes a meal ‘healthy’ to you (post-its on the map)
- Group discussion – recruitment and engagement strategies
- Use whiteboard
- How/where to reach families for research related to food insecurity
- Communication ecology
- Language around ‘food insecurity’
- Communication pathways with families

**Workshop run sheet: 90 minutes**

**Welcome & Introductions –** overview of the purpose of the workshop

**Activity 1: Warm-up activity**

Close your eyes and draw a self-portrait; show your portrait and tell the group 2 things about yourself (facilitators participate in the activity)

**Activity 2: Mapping food-related everyday decisions:**

***Part 1***

- In this activity, we'll break down the steps to prepare a typical family meal. By identifying the important decisions at each step, we can better understand the challenges and opportunities we face in providing meals for our families.
- Consider your family’s food and meals.

As a group, let’s take a practical journey to map the process of putting food on the table. We will explore each step, from the initial decisions about what to eat to getting the food, storing, preparing, and eating food at home. (use the whiteboard and map this together)

- Now complete this journey map for your family with two situations. The first is a meal at home on a good week, say at the beginning of your pay cycle. For each step, think about things or strategies you do that make this easier or harder. This could include time spent, effort, and mood for each step. Think about the things around you that make this easier or harder, e.g., the location of shops, transport, and caring for kids. Now think about making a meal for the family when things are harder, say at the end of your pay cycle or if you’ve had an unexpected bill come up. How is this different?

***Part 2:***

- Use markers/post-its or highlighters to mark these things on your map – use green for things that make each step easier, red for what makes each step harder, and yellow for neutral, or it could go either way.
- Discuss as a group – look at similarities/differences between families.

***Part 3:***

What is a healthy meal to you? On the group journey map or their family map

1. Looking at the food map again, is the meal at the end healthy? Why/Why not?
2. What factors in the journey map influence whether the meal is healthy?

**Activity 3: Recruitment and Engagement Strategies:**

- Brief overview of the planned research and parent involvement at different stages (on whiteboard/ paper)

Group discussion:

How do we reach families that may be food insecure?

How do we communicate and keep in touch?

Framing of ‘food insecurity’: what is the right language?

Where and how would families find out about our research?

**Communicative ecology:** What is the best way to communicate with families? How do you communicate with others about food-related topics?

How do we keep families engaged throughout the research?

What would be suitable incentives?

How can we share the outcomes of the research with families?

How can we help families and organisations work together and exchange ideas?

**Wrap up, summarise key talking points from the workshop and invite comments.**

**Thank you!**

**Workshop 2: Support & Information Mapping**

**Resources:**

- Consent forms
- pens and markers
- facilitator checklist - admin
- workshop guide
- Templates – House, Baby
- Large paper sheets
- Individually wrapped snack items
- Thank you vouchers
- emoji stickers

**Activity overview:**

- Family support network
- Parenting Resource map
  - Create a visual map of your experiences accessing parenting information, formal and informal. Label those experiences positively or negatively.
  - Include Social/tech/media mapping
- Baby hunger and fullness cues

**Workshop run sheet: 90 minutes**

**Welcome & Introductions –** share the purpose of the workshop

**Activity 1: Warm-up**

*Share a piece of advice that you would give to a friend who is expecting their first baby. It might be something useful that you have heard or experienced for yourself.*

- Write on the whiteboard [take a photo and create a word art to share via email after the session– confirm the group is happy to receive this]

**Activity 2: What does my family look like**? (use Template_W1 Activity 2_A3 size)

*All families are different; this activity is about getting a picture of your family and who supports your family.*

*Looking at the house here [hand out the template of a house], write who is in your household, inside the house. Around the house, put down the people who support you and your family, especially with feeding your family. These are your support crew, the people who help you out when you need it. This can be family, other mums or parents, the person who runs a parent group or playgroup, someone at your church, your GP…*

*Take a few moments to do that, feel free to draw stick figures, label them, or write them out.*

**Activity 3: Create a Feeding Resource Visual Map**

*The next activity is about where and how you get information, help, or advice about feeding your baby or toddler. This can be from places like Facebook or social media, a friend, or health professionals. What we want to capture here is all the experiences you’ve had, both good and bad.*

- Use large butcher paper and ink pens - ask participants to brainstorm sources of feeding info/advice and label
- Use Post-it notes and write directly on the sheet, use lines to make connections

Prompts

- - What about programs you have done in the past? e.g. antenatal classes? Child health or mothers’ groups? Introduction to solids?
  - Social media technology and entertainment?
  - Health professionals – e.g. GP, child health nurse, early childhood educator, pharmacist/pharmacy assistant.
  - Cost – were these resources free to you, what sources cost money?
  - Think about the experiences your friends and family shared with you.

*We also want to capture negative experiences here, not just those you would recommend to others.*

*Now, for the fun part, here are some emoji stickers. Let’s go through all these and label them with emoji stickers to represent that experience – was it good, frustrating, or maybe a crying emoji?…. Feel free to add some words to explain if you would like to*

- - use the emoji stickers to label touchpoints as good or bad; ask participants to add some words to say why (or give an example}
  - parents might have different experiences with the same source of support
- Discuss as a group and allow this to lead to parent’s thoughts on the best sources of child-feeding support

**Last Activity - Hunger/Full Baby**

A3 Template of a drawing of a sitting baby with one Hungry and the other saying Full

*Think about how you know or knew when your baby or toddler was full or hungry*

- Let’s put this up on the sheet on our ‘ full ‘ and ‘ hungry ‘ baby

**Wrap up, summarise key talking points from the workshop and invite comments.**

**Thank- you!**

**Workshop 3: Intervention Messaging/Language & Modality**

**Resources:**

- Consent forms
- pens and markers
- facilitator checklist
- workshop guide
- Templates – Baby persona, key messages with ratings
- Large paper sheets
- Individually wrapped food items
- Thank you vouchers

**Activity overview:**

1. Baby Persona (leading to what is responsive feeding/why responsive feeding)
2. Responsive feeding key messaging –feedback on tone/language/phrasing and what to change
3. Create a Parenting resource map
4. Brainstorm activity for our responsive feeding parent program - how would you want this to look? Modality/timing/intensity/ duration

**Workshop run sheet: 90 minutes**

**Welcome & Introductions –** overview of the workshop’s purpose

**Activity 1: Warm up – ‘Baby persona’**

*If you are comfortable, close your eyes. Imagine…..you exist in a warm, safe space that is constant in all sensory things like sound, temperature and pressure. It is calm, predictable, and safe…. Take a moment to imagine what this experience would be like….Then suddenly, you are flung into a constantly changing world, loud, intense, and scary at times, and there is so much to learn….*

*What’s going on here?*

- Invite some comments on what parents think their babies might have said in their first months if they could talk. Remember a particular look from your baby? What do you think your baby was trying to tell you?
- Now, consider the experience of learning to eat from a baby's point of view. Is it pleasant? Is it fun, or is it stressful?

Baby persona activity…. Use the ‘baby’ template. Invite participants to write or use thought bubble stickers to write inside—characteristics, experiences, feelings, thoughts—use feeding as the scenario.

- Invite participants to share their thoughts on this activity.

*Use this activity as a lead-in to discuss responsive feeding and what it is and looks like.*

- Invite comments.
- discussion point—now that we’ve discussed responsive feeding, what do you think non-responsive feeding would look like when feeding a baby or child?

**Group Reflection**

*Thinking about yourself – do you eat to your appetite? The amount of food your body needs to be full and healthy but not more.*

- Discuss people's experiences as a group

We were all born with an internal ability to know how much food to eat. Adults often lose this ability and eat for reasons other than hunger, like being stressed or sad or from the habit of finishing their plate. Often, these habits come from how we were fed growing up.

**Activity 2: Feedback on Responsive feeding messaging**

*Let’s explore some key messages from a previous child-feeding program that was developed for parents.*

Key program messages/principles:

- The way we feed young children affects the foods they will like and their health
- Learning to like and like to eat. Children need to be offered a food up to ten times to learn to like the taste.
- Listen to and trust your child. ' Parents provide, children decide’—babies and children decide how much to eat.
- Early habits track to adulthood. Offer children a variety of foods as taste preferences are developed early
- Set good examples for your child. Eat healthy foods in front of your child
- Your relationship with your child is important

***Part 1 – Activity***: Rating of key messages

[Posters of the key messages are pinned up around the room; invite participants to move around the room and rate the messages]:

(scale used is a line: not at all -------------- very much)

- - Do you agree with this?
  - Does this make sense to you?
  - Are you likely to do this in your house?
  - Is this important to you?

***Part 2 - Discussion***: Let’s look at each key message as a whole group.

- Invite parents to share their responses to the above questions and their thoughts on the messages—did you like/not like them? Which ones/why?
- Invite suggestions for how to adapt them – different wording?

**Activity 3: Parenting Resource Map & Brainstorming activity**

[Facilitator’s draw on workshop 2, Activity 3 Resource map]

*Think about any parenting and child health programs you have done – it could have been face-to-face groups, digital information, an app, a book*

- Let’s map these on the poster - how was the parenting information delivered.
- What parenting programs / informational resources have you experienced before?
- What did you think worked well for you/ what didn’t?
- What other ways would you want to learn about parenting or child feeding? [Facilitator to add items from previous workshop and share with the group]

**Brainstorm ideas for intervention.**

Now, consider responsive feeding. What are your thoughts and ideas on how best to promote this approach to parents?

- Prompt on intervention mode, structure, intensity, timing etc.
- Be creative – there are no limits here, and you don’t have to be practical.
- Discuss as a group

**Wrap up, summarise key talking points from the workshop and invite comments.**

**Thank you!**
