# Supplementary material for: A design thinking‐led approach to develop a responsive feeding intervention for Australian families vulnerable to food insecurity: Eat, Learn, Grow
Source: Health Expect. 2024 Apr 20;27(2):e14051. doi: 10.1111/hex.14051 (PMC11032130; doi:10.1111/hex.14051)
Supplement: Supplementary file 3 — Supporting information. [file HEX-27-e14051-s003.docx]

# Appendix 3: The Responsive Feeding in Tough Times Project: Codesign workshop protocol and guide – Health Professionals (workshop 4)

**Workshop: Expert Content Review**

**Resources:**

- Consent forms
- pens and markers, post-it notes
- facilitator checklist - admin
- PowerPoint slides
- templates needed – mock-up of intervention mail-out pack contents; A3 books of intervention program i.e., screenshots of digital modules and scripts with QR codes
- IT resources for Zoom link for remote attendees
- Participants to BYO mobile phone device with headphones (advised in advance)
- Fruit platter

**Activity Overview:**

- Overview of research program
- Describe the developed intervention, study design and evaluation plan
- Content Review Activity
- Group discussion

**Workshop run sheet: 2 hours**

**Welcome & Introductions –** overview of the purpose of the workshop

**Activity 1: Orientation to the research program, objectives, and guiding framework**

- Provide a brief presentation of the research program, objectives, and a description of the guiding framework used – Design Thinking

**Activity 2: Overview of findings and intervention development**

- Provide an overview of the findings of the engagement work undertaken with parents
- Share preliminary findings and research outputs
- Provide an overview of the developed intervention – Eat, Learn, Grow
- Demonstrate mock-up of mailout package of resources co-designed with parents

Group discussion on the preliminary findings shared and intervention design, invite comments and questions.

**Activity 3: Expert Content Review**

Direct participants to the intervention content book with screenshots of digital modules with QR codes [hand out A3 books]

- Provide an overview of the principles of Plain Language writing and design of materials.

Question prompts to consider [provide on a slide which stays up during activity]

Is the content safe, appropriate, and congruent with responsive feeding theory?

Does the writing avoid the use of jargon and use Plain Language principles?

Do the imagery and videos represent adequate diversity? – Dads, ethnicities, mealtime settings

Does everything work? - The interactive elements, the padlets

Does it have the right ‘feel’? – contextual for hardship/low income, reassuring, non-judgemental

Are there any typos?

Could it be better?

- Invite participants to review the content of the intervention. Ask participants to get comfortable and to use their mobile phone device with headphones to engage with the content as intended for parents by scanning the QR codes.
- Direct participants to review the content in the order the modules are presented in the book, as this reflects how parents will receive the digital modules.
- Ask participants to make notes, use post-its and mark up any comments or suggestions for change on the content books in the spaces provided and to draw lines to show where their feedback relates to the module content
- Remind the participants to view the scripts when listening to all audio and video content and mark up these scripts with any feedback.

**Activity 4: Debrief Discussion of feedback**

- Invite feedback and discussion
- Allow time for the intervention structure, study design, and evaluation plan to be reviewed.

**Wrap up, summarise key talking points from the workshop and invite comments.**

**Thank you!**

Ensure all booklets with feedback are collected.
